# Myocardical Infarction in Young Adults: Revisiting Risk Factors and Atherothrombotic Pathways

**DOI:** 10.3390/medicina61091615

**Published:** 2025-09-07

**Authors:** Petre Alexandru Cojocaru, Maria Loredana Țieranu, Mina Teodora Luminița Piorescu, Ionuț Cezar Buciu, Alexandru Mugurel Belu, Silvana Isabella Cureraru, Eugen Nicolae Țieranu, Gianina Cristiana Moise, Octavian Istratoaie

**Affiliations:** 1 Department of Cardiology, University of Medicine and Pharmacy of Craiova, 200349 Craiova, Romania; 2 Department of Obstetrics and Gynaecology, Emergency County Hospital, 200642 Craiova, Romania; 3 Department of Physiology, University of Medicine and Pharmacy of Craiova, 200349 Craiova, Romania; 4 Department of Dermatology, Emergency County Hospital, 200642 Craiova, Romania; 5 Department of Cardiology, Emergency County Hospital, 299642 Craiova, Romania

**Keywords:** young adult, myocardial infarction, risk factors

## Abstract

*Background*: Myocardial infarction (MI) in young adults, once a rarity, is increasingly recognized as a distinct clinical entity. Unlike traditional MI patients, younger individuals often present without established risk factors or advanced atherosclerosis, prompting a reevaluation of pathophysiologic paradigms and risk assessment strategies. *Objective:* This review synthesizes current evidence on the epidemiology, pathophysiology, and diagnostic challenges of MI in adults under 55 years, with emphasis on risk factor profiles. We distinguish between traditional cardiovascular risk factors—smoking, dyslipidemia, hypertension, diabetes, obesity, and family history—and emerging contributors, including elevated lipoprotein(a), recreational drug use (cocaine, cannabis, amphetamines), autoimmune and inflammatory conditions, psychosocial stress, sleep disorders, genetic predisposition, and non-atherosclerotic mechanisms such as myocardical infarction with non-obstructive coronary arteries, spontaneous coronary artery dissection, SCAD and Takotsubo syndrome. *Methods:* A narrative literature review was conducted, focusing on studies from the last five years addressing MI in young adults, including data from large registries, cohort studies, and recent experimental findings.

## 1. Introduction

Traditionally considered a condition of middle-aged and older adults, Myocardial infarction (MI) is increasingly affecting younger individuals—often in the absence of conventional risk factors such as long-standing hypertension, diabetes, or established atherosclerosis. This emerging trend raises important questions about the adequacy of existing cardiovascular risk models and suggests the presence of alternative, underrecognized contributors to early-onset atherothrombotic disease.

In recent years, attention has turned to a range of novel risk factors that may play a disproportionately large role in the pathogenesis of MI in young adults. These include genetic variants (e.g., elevated lipoprotein(a)), inflammatory and autoimmune processes, thrombophilic states, the use of recreational substances (notably cocaine and cannabis), and persistent psychosocial stress. Lifestyle patterns unique to younger populations—such as sleep deprivation, disordered eating, and digital-era stress—also appear to exert independent cardiovascular effects.

Moreover, sex-specific aspects, especially the increasing recognition of MI in young women—often presenting without obstructive coronary disease—further complicate the clinical picture and highlight the limitations of traditional diagnostic and preventive strategies.

Given the potentially devastating socioeconomic consequences of MI of young people there is a pressing need to better characterize the spectrum of risk factors in this population. Such understanding will inform targeted prevention and long-term strategies for this.

This review explores the evolving landscape of MI in young adults, focusing on the expanding spectrum of risk factors and pathophysiological mechanisms. By reevaluating current paradigms and integrating emerging evidence, we aim to better understand, identify, and ultimately prevent premature cardiovascular events in this unique population.

## 2. Epidemiology of Premature Myocardic Infarctions

In recent years, the clinical landscape of MI has undergone a noteworthy demographic shift. Once considered predominantly a disease of older adults, MI is now increasingly observed in younger individuals—often in their 30s or 40s—who may lack long-standing chronic conditions traditionally associated with coronary artery disease. This evolving epidemiological profile has drawn growing attention in cardiovascular research and practice [1].

Current estimates suggest that approximately one in ten patients hospitalized for acute MI is under the age of 55. While men continue to represent the majority of these cases, recent data highlight a worrying trend: the incidence among young women appears to be rising or, at the very least, not declining at the same rate. Compounding this, women are more likely to present with atypical symptoms, experience delays in diagnosis, and suffer worse in-hospital outcomes [2].

Geographical and socioeconomic differences play a significant role in the distribution of premature MI. In some high-income countries, incidence rates have plateaued or slightly decreased, largely due to better primary prevention. However, in many low- and middle-income regions, the number of young adults affected by MI remains substantial and, in some cases, continues to grow—reflecting disparities in healthcare access, education, and exposure to modifiable risk factors [3].

Although traditional cardiovascular risk factors such as tobacco use, hypertension, hypercholesterolemia, and diabetes are still prevalent among young MI patients, they often coexist with newer, underrecognized contributors. Emerging evidence implicates psychosocial stress, recreational drug use, sedentary behavior linked to modern work environments, and proinflammatory conditions in the early development of atherothrombotic events. In addition, genetic susceptibilities—such as elevated levels of lipoprotein(a) or familial hypercholesterolemia—may play a disproportionately large role in this group, especially in the absence of overt lifestyle-related risk [4].

The rising incidence of MI in younger populations, combined with their unique clinical characteristics and risk profiles, underscores the limitations of current cardiovascular risk assessment tools, which were largely developed based on older cohorts. Addressing this growing challenge requires not only earlier identification of at-risk individuals but also a broader understanding of the multifactorial nature of premature coronary disease—one that integrates biological, behavioral, environmental, and social dimensions [5].

## 3. Global Incidence and Prevalence

Determining the actual prevalence of coronary artery disease (CAD) in young adults remains a significant challenge, mainly because the clinical characteristics of both atherosclerotic and non-atherosclerotic forms are still not clearly delineated. This is particularly evident in cases of myocardial infarction with non-obstructive coronary arteries (MINOCA), where the absence of standardized diagnostic protocols and limited use of intracoronary imaging often results in misdiagnosed or overlooked non-plaque mechanisms [6].

Available epidemiological data on MI in younger individuals are sparse. Findings from the Framingham Heart Study illustrate a steep age-related increase in MI incidence among men—from 12.9 per 1000 in those aged 30–34 to 71.2 per 1000 in the 45–54 group. Women show consistently lower rates across the same age brackets. Notably, over a quarter of the myocardial infarctions in this study were asymptomatic, with unrecognized events occurring more frequently in women [7].

In South Asia, especially in India, early-onset CAD presents another layer of complexity [8]. Data from a cohort of 877 patients indicated that roughly a third were diagnosed before turning 45. In over 90% of these cases, common lifestyle-related risk factors were present, including low fiber intake, smoking, hypertension, diabetes, dyslipidemia, alcohol use, sedentary habits, psychosocial stress, and central obesity. Notably, risk profiles differ by gender: young women with MI tend to have higher rates of comorbidities like hypertension, diabetes, depression, and heart failure, whereas men are more often affected by physical inactivity and elevated cholesterol levels. Evidence from the VIRGO and GENESIS-PRAXY studies suggests that young women may experience MI through less well-understood mechanisms, recover more slowly, and face higher risks of complications, readmission, or death compared to men their age [9,10].

Although the overall incidence of coronary heart disease (CHD) has declined in the UK, the trend does not necessarily reflect what is happening among younger adults. Data between 1992 and 2012 show very low CHD rates in the 35–44 age group—0.5% in men and 0.18% in women—but prevalence increases sharply in older age groups. Younger individuals are likely underdiagnosed, due to less typical symptom presentation and lower rates of medical consultation. As a result, only 3% of all CHD diagnoses are made in people under 40, a figure that may underrepresent the true burden in this demographic [11,12].

Modifiable risk factors remain central to the rising trend in early-onset CAD. Smoking, in particular, is widespread among young adults, with rates climbing to nearly 10%. Young women in the UK have been reported to smoke more heavily and for longer durations, potentially diminishing the protective cardiovascular effects of estrogen [13]. In parallel, obesity has surged among children and young adults, with rates tripling in the last two decades. Cocaine use, another major concern, is a recognized precipitant of chest pain and myocardial infarction in younger people and continues to be frequently implicated. Taken together, these patterns suggest that while CAD may appear to be declining in the general population, a significant and growing risk is emerging among younger individuals—one that warrants much closer attention (Table 1) [14,15].

This table summarizes the main cardiovascular risk factors in young adults, distinguishing between traditional and emerging contributors. While traditional factors remain highly prevalent, emerging risks are increasingly recognized as significant drivers of premature MI.

## 4. Traditional Risk Factors

**Figure 1:**
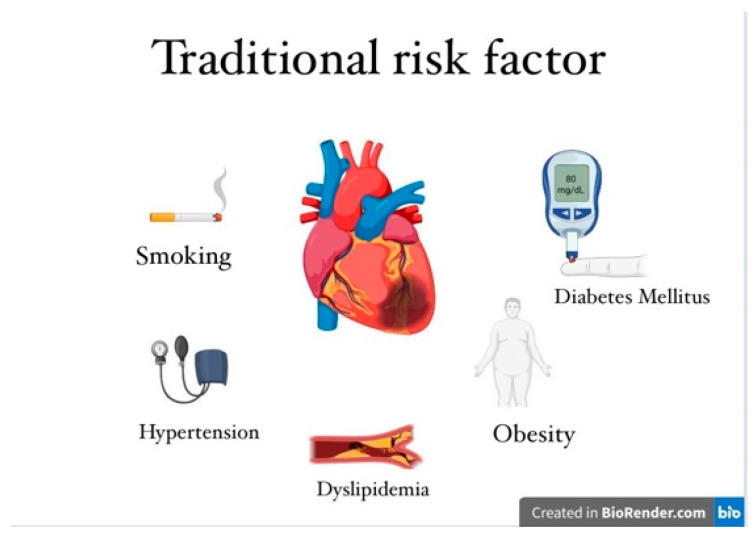
Traditional and novel cardiovascular risk factors in young people.

Young patients with myocardial infarction share traditional cardiovascular risk factors with older individuals, including smoking, obesity, hypertension, diabetes mellitus, and dyslipidemia. However, a significant proportion of these younger patients differ from their older counterparts in terms of emerging risk factors. Young patients often use recreational drugs and may present with autoimmune diseases, familial hypercholesterolemia, elevated lipoprotein levels, or psychosocial factors such as stress, depression, and burnout—all increasingly recognized contributors to myocardial infarction in this age group (Figure 1) [14,15].

### 4.1. Dyslipidemia

Dyslipidemia is an important risk factor in young people who suffer with acute myocardical infarction AMI, with over 80% having at least one abnormal lipid along other risk factors [5]. Some studies data show that lipid profiles have improved in some European young adult populations but AMI in these patients continues to rise [4].

In a cohort of adults under 40 age who suffered an AMI, 40 percent of them had abnormal levels of lipid, most commonly hypertriglyceridemia, high low density lipoprotein cholesterol LDL-C and low high density lipoprotein cholesterol HDL-C [16].

A Korean study found that statin use was associated with a higher AMI risk compared to non-use. Non-statin users who had LDL-C > 120 mg/dL faced a 33% higher AMI risk than those with <80 mg/dL, while statin users with LDL-C < 80 mg/dL had a 66% higher risk than non-users with similar LDL-C values. Moreover, HDL-C remains a consistent risk marker in younger adults [16,17,18,19].

### 4.2. Hypertension

Hypertension contributes significantly to the development of atherosclerosis in the young. While often asymptomatic, elevated blood pressure is frequently underdiagnosed and untreated in this age group, exacerbating cardiovascular risk [20]. Results show that young people received a diagnosis slower compared to older patients.

Delayed diagnosis allows vascular damage to progress silently, amplifying long-term cardiovascular risk [21].

Globally, the prevalence of undiagnosed hypertension is high. An Indonesian study revealed that 55% of men and 44% of women age 26–35 age were undiagnosed [22].

### 4.3. Smoking

Cigarette use promotes endothelial inflammation and dysfunction followed by thrombosis and lipid oxidation, accelerating atherosclerosis without an important plaque burden.

Smoking remains one of the most common risk factors for AMI among all people. In a recent study, 52.5% from individuals were smokers and in a Danish register data of patients, age 30–49, 74% were current smokers compared with the rest of traditional risk factors (10% hyperlipidemia, 15% hypertension, 7% diabetes). Recent studies reveal that it increases the risk of AMI by 9-fold in men and up to 13-fold in women [14].

Multiple studies confirm that active smoking is a stronger cardiovascular risk factor for younger women than for men. A UK cohort study revealed that women had a 13-fold higher risk of AMI compared to an 8.5-fold risk in men [23].

Smoking cessation also plays an important role: young people who quit smoking within one year after AMI had a 70% reduction in all-cause mortality and an 80% reduction in cardiovascular mortality over an 11-year follow-up. [14,24]

### 4.4. Obesity

Recent studies have shown a strong association between obesity and AMI in young people. Data from numerous studies of AMI reveal that obesity is present in 78% of the patients suffering from AMI at a young age. In young women, a BMI > 30 increased the risk of AMI nearly by 4.7-fold (HR 4.71, 95% CI: 3.88–5.72) and was also associated with higher cardiovascular mortality [25,26].

### 4.5. Diabetes Mellitus

Diabetes mellitus is an important modifiable risk in young people suffering from AMI. A study from 2024 reveals that glycemic variability in patients with AMI is correlated with worse outcomes and higher rate of in-hospital mortality. The mortality of patients who suffer from diabetes mellitus with high glycemic values is increased by 1.25–3.40 [27]. Glycemic fluctuations were associated with higher in-hospital mortality, particularly among patients with AMI whose admission blood glucose was within the normal range.

## 5. Emerging Risk Factors 

### 5.1. Recreational Drug Use

**Figure 2:**
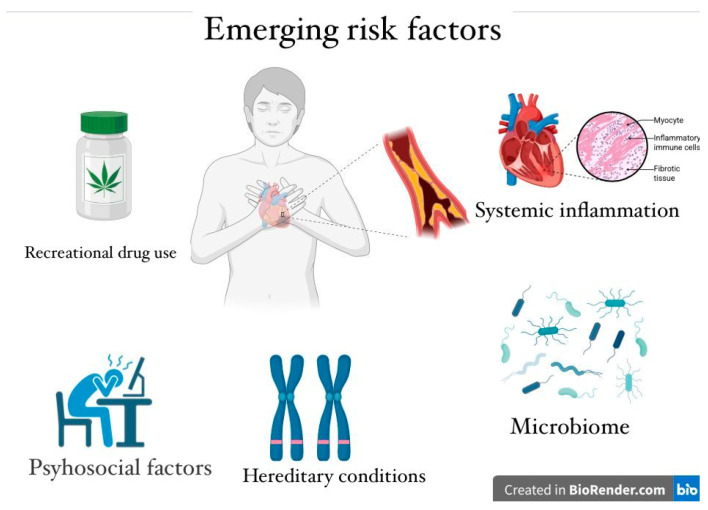
Emerging cardiovascular risk in young people.

In recent years, recreational drug use, particularly cannabis and cocaine, has been recognized as an independent risk factor for AMI among young people (Figure 2) [28].

Cocaine stimulates alpha-1 and beta-1 adrenergic receptors, increasing heart rate and systemic blood pressure, which in turn elevates myocardial oxygen demand. It can also induce coronary vasospasm hours after use, particularly in epicardial vessels, reducing myocardial oxygen supply. Furthermore, it activates platelets and plasminogen, contributing to thrombus formation [29,30,31]. Chronic and acute uses have a different impact on the cardiovascular system. While chronic consequences remain less defined, the acute cardiovascular effects of cocaine have been extensively documented. As a powerful stimulant, cocaine use has been linked to electrocardiographic changes, heightened blood pressure, arrhythmias, and AMI. The likelihood of MI in cocaine users is shaped by both underlying cardiac risk factors and high-risk behaviors. Mechanistically, cocaine can provoke acute events through multiple pathways, including inhibition of cardiac sodium and potassium channels and promotion of coronary artery spasm or vasoconstriction. By contrast, the long-term cardiovascular effects of cocaine remain less clearly defined, with previous studies reporting inconsistent results [29].

Cannabis increases heart rate and blood pressure and contributes to platelet aggregation, endothelial dysfunction, and coronary vasospasm.

In AMI registries, 10.7% of young patients reported cocaine and/or cannabis use. A French study found that 12.6% of patients with AMI tested positive for drug use, 34% of whom were under 50 [32,33].

### 5.2. Systemic Inflammation and Autoimmune Disease

Systemic inflammation and autoimmune disease make an important impact on the patient’s life, with an even greater effect in young patients suffering from AMI.

Young AMI registry data reveal that 2.5% of patients under the age of 50 had had an autoimmune disease or systemic inflammation (SID), such as rheumatoid arthritis, psoriasis, systemic lupus erythematosus, or multiple sclerosis. These patients were more often women and had hypertension compared to others. Patients under age 50 with SID had more than twice the all-cause mortality over 11 years [34].

Rheumatoid arthritis (RA) is a chronic autoimmune disease that increases cardiovascular (CV) mortality by up to 50%, with a risk of acute coronary syndromes comparable to type 2 diabetes [35]. Beyond traditional factors, persistent systemic inflammation drives endothelial dysfunction, accelerates atherosclerosis, and promotes unstable plaque formation. The distinct CV profile in RA includes myocardial infarction, sudden death, and silent ischemia [36]. The “lipid paradox”—low lipid levels despite high CV risk—further reflects the central role of inflammation, but targeted anti-inflammatory therapy can reduce this burden [37].

In systemic lupus erythematosus (SLE), young patients—particularly women under 45—carry a disproportionately high risk of myocardial infarction, with incidence rates up to ten times greater than in the general population [38]. This vulnerability is fueled not only by conventional risk factors such as hypertension and dyslipidemia but also by disease-specific drivers: persistent systemic inflammation, immune-mediated vascular injury, high disease activity, long-term corticosteroid exposure, and antiphospholipid antibodies. These mechanisms accelerate atherosclerosis, destabilize plaques, and heighten thrombotic potential, enabling severe coronary events to occur even in the absence of traditional risk profiles. Prompt control of inflammation and proactive cardiovascular surveillance are therefore critical to reducing the burden of premature AMI in SLE [38].

In the last 5 years, multiple cohort studies and meta-analyses have confirmed that young adults with ankylosing spondylitis (AS) or axial spondylarthritis face a significantly higher risk of myocardial infarction, even after adjusting for traditional factors [39]. Persistent systemic inflammation (TNF-α, IL-17), endothelial dysfunction, arterial stiffness, and frequent NSAID use accelerate atherosclerosis, while metabolic syndrome and smoking amplify this risk. TNF inhibitors appear protective [40], with real-world data linking effective inflammation control to fewer acute coronary events. Early cardiovascular screening and aggressive disease management are essential to reduce premature MI in AS patients.

Psoriasis is a systemic, immune-mediated disease that accelerates atherogenesis and raises ASCVD risk beyond traditional factors, with signal strongest in moderate–severe skin disease. Mechanistically, chronic IL-17/TNF-α–driven inflammation promotes endothelial dysfunction, arterial stiffness, and pro-thrombotic pathways—biologic plausibility that aligns with higher MI/ACS rates in observational cohorts and contemporary reviews [41]. Recent evidence links disease severity and duration to coronary microvascular dysfunction—an early substrate for type 1 MI—supporting the concept of “premature” coronary disease in younger patients with long-standing psoriasis [42]. Therapeutically, large, real-world, meta-analytic data suggest patients treated with modern biologics (anti-TNF, anti-IL-17, anti-IL-23) experience lower incident cardiovascular events versus oral/non-biologic therapy, consistent with the inflammation-hypothesis; signals are not uniform across all agents (e.g., anti-IL-12/23) [43].

Medium–large-vessel inflammation can directly involve coronaries—causing ostial stenoses, aneurysms, thrombosis, and rapid restenosis—on top of diffuse endothelial injury and pro-thrombotic signaling. The result is acute coronary events at ages where atherosclerosis alone would be unlikely. Takayasu arteritis (teens–young women): Contemporary reviews show frequent coronary involvement (esp. ostial/proximal lesions) with MI as a major cause of TAK-related death; multimodality imaging refines detection [44]. Kawasaki disease (KD) (childhood) → young-adult MI: Persistent or giant coronary aneurysms carry long-term atherosclerotic change and MI risk years after the acute illness [45]. ANCA-associated vasculitis (AAV): Recent cohort/meta-analytic data show substantially higher cardiovascular events, including MI; risk peaks in the months after diagnosis and remains elevated [46]. Polyarteritis nodosa (PAN): Medium-vessel necrotizing vasculitis with documented coronary arteritis/occlusions and MI—even in young patients—though uncommon overall [47].

Systemic inflammation can profoundly affect lipid metabolism, exemplified by the paradoxical LDL-C rise seen after inflammation control in rheumatoid arthritis. In the YOUNG-MI registry, patients with SID had similar overall lipid profiles to those without SID but showed a tendency toward higher triglycerides (*p* = 0.04). Despite comparable renal function at presentation, SID patients had significantly lower peak troponin levels during the index MI (*p* = 0.003), suggesting possible differences in myocardial injury patterns [34,48].

Studies also reveal that autoimmune diseases contribute to AMI risk though endothelial dysfunction caused by inflammation, the immune complex deposition, and the prothrombotic states [34,49].

Among inflammatory markers, high-sensitivity C-reactive protein (hsCRP) has uniquely transitioned into routine cardiovascular risk assessment, reflecting hepatic response to IL-6 stimulation. While CRP itself is not a causal driver of atherothrombosis, upstream mediators such as IL-1 and IL-6 actively promote plaque development and destabilization. Other candidates—myeloperoxidase (MPO), lipoprotein-associated phospholipase A_2_ (Lp-PLA_2_), and trimethylamine-N-oxide (TMAO)—highlight diverse inflammatory and metabolic pathways linking immune activation to vascular injury. MPO fosters oxidative modification of LDL and impairs HDL function, Lp-PLA_2_ amplifies oxidative stress within plaques, and TMAO, generated from gut microbial metabolism of red meat and eggs, accelerates foam cell formation, platelet activation, and thrombosis. Despite strong mechanistic evidence, these markers remain largely research tools; their integration into everyday risk stratification for young patients with AMI awaits effective targeted interventions and broader validation across populations [50,51].

The presence of post-AMI autoimmune responses, such as Dressler syndrome, highlights the importance of autoimmune system responses against myocardial neo-antigens formed as a respond to AMI [52].

### 5.3. Hereditary Conditions: Hypercholesterolemia and Lipoprotein(A)

Genetic lipid disorders play an important role in the development of early AMI, underscoring the importance of early diagnosis to enable timely treatment and prevention.

Familial hypercholesterolemia has a high prevalence among young patients with AMI, as 1–5% are heterozygous for familial hypercholesterolemia (compared to 0.5% in the rest of population). This condition increases the risk of AMI at a young age by 15–20 times compared to patients without familial hypercholesterolemia, and affected patients have a 2.3-fold higher risk of recurrence. A study of 690 patients revealed that those with familial hypercholesterolemia were more likely to have three-vessel disease (*p* = 0.007), as well as a higher thrombus burden and final TIMI slow/no-flow (*p* = 0.027) compared with patients unlikely to have the disease (*p* = 0.006) [53,54,55].

**Figure 3:**
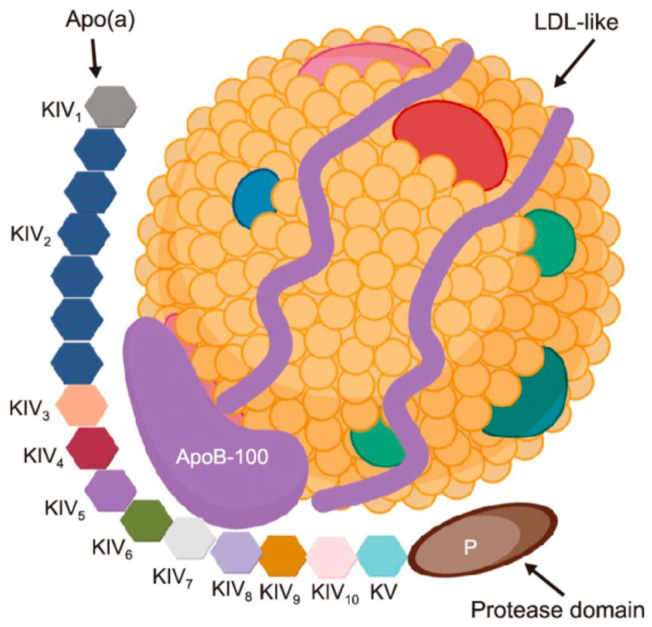
Structure of lipoprotein(a). Created with BioRender.com. version 4.0.

Lipoprotein A (LpA) (Figure 3) is a stronger independent risk factor in young people with AMI due to pro-atherogenic and pro-thrombotic properties, as it carries cholesterol molecules. High levels of LpA are correlated to 2–3-times higher risk of AMI compared to normal levels. Moreover, levels > 50 mg/dL affect 20–25% of people worldwide, and a value more than 180 mg/dL place the patients at a very high risk of AMI. A U.S. multi-ethic cohort revealed that high levels of lipoprotein(A) is linked to a very high risk of AMI. In the future, this lipoprotein may be a screening test for preventing AMI in patients of any age [56,57,58,59,60].

Other genetic lipid disorders implicated in AMI at a young age include ApoA5 polymorphism, which is linked to elevated triglyceride levels and thereby increases AMI risk, also serving as a predictor of early AMI [56,58,61].

### 5.4. Psychosocial Factors: Stress, Depressions, Burnout

Increased responsibilities, academic and career pressures, and social isolation contribute to rising psychological distress in younger populations.

Meta-analyses found that depression has a 24% prevalence in patients suffering from AMI, 12% have anxiety, and 10% of them suffer from PTSD. Early psychological intervention has a very important role in the prevention and recovery to those suffering from AMI [62,63].

### 5.5. Endothelial Dysfunctions and the Microbiome

The idea that microbiome is an important factor in the development of cardiovascular diseases has gained traction in the last decade due to the influence of the metabolic system. The microbiome influences trimethylamine N-oxide, short-chain fatty acids, and bile acid pathways, contributing to heart failure, atherosclerosis, hypertension, myocardial fibrosis, and coronary artery disease [58,64].

Viral (e.g., cytomegalovirus, influenza A, hepatitis C) [65] and bacterial (e.g., Chlamydia pneumoniae, Helicobacter pylori) infections have been implicated in atherosclerosis via endothelial activation, cytokine upregulation, and heat shock protein expression [66,67]. While infection-driven inflammation is a plausible contributor, evidence from the Tsimane population—with high hsCRP prevalence but minimal coronary calcification—suggests that inflammation alone, in the absence of conventional risk factors, may be insufficient to promote coronary artery disease [68].

A 2025 study found that patients with AMI had blood samples enriched in Firmicutes, Bacteroidota, Actinobacteriota, and Bacteroides; however, the clinical significance of those in AMI remains unclear and requires further investigation [64].

Dysbiosis, including reduced microbial diversity and a shift toward Bacteroides-dominant profiles, may promote myocardial fibrosis and coronary artery disease. Further studies are needed to clarify the microbiota’s role in AMI [69].

HIV infections play an important role in AMI in young patients. Despite modern antiretroviral therapy, young people living with HIV face a 1.5–3× higher risk of myocardial infarction than HIV-negative people, often occurring a decade earlier. The culprit is not simply atherosclerosis but a triple hit [70].

1. Immune dysregulation: Persistent monocyte/macrophage activation and cytokine release (IL-6, TNF-α) destabilize plaques even when viral load is undetectable [70,71].
2. Endothelial injury: HIV proteins and chronic inflammation drive microvascular dysfunction, creating fertile ground for both type 1 and type 2 MI [71].
3. Therapy-related effects: While integrase inhibitors are generally safer, certain regimens, especially recent abacavir exposure, have been linked to abrupt rises in MI risk [71].

## 6. Clinical Implication

Typical symptoms are chest pain with or without radiation to the arms or jaw, diaphoresis, dyspnea, and dizziness. Atypical symptoms are digestive symptoms like epigastric pain, indigestion, nausea, or vomiting, as well as neurological symptoms like fatigue, neck or back pain, or even syncopae. These are more often present in young women, with up to 90% experiencing atypical symptoms [72].

Some meta-analysis found that 11.6% of all patients suffering from AMI are presenting with atypical symptoms and 33.6% are having no chest pain. Small studies reveal that 69% of young patients with AMI have no chest pain before the event [72].

These atypical symptoms of presentation can lead to misdiagnosis, increasing in-hospital mortality (19% in atypical symptoms vs. 3% in typical symptoms) due to the delayed time of intervention; as a result, it is essential to know the non-specific symptoms for more rapid interventions [73].

The variation in symptomatology is related to neural modulation and to acute inflammatory or ischemic changes caused by epicardial coronary artery ischemia [74].

Due to the atypical symptoms on presentation, diagnosis is often delayed, and the time to PCI is often longer.

A South Asian registry showed that young patients with AMI have 125 min longer waiting time of the medical service presentation compared to elderly patients, and a European study revealed that the uncertainty diagnosis in primary care led to longer time of waiting for younger patients [75].

Japanese studies revealed that these problems persist, and as a result, patients fail to meet the guideline-recommended benchmarks for early intervention: <10 min door-to-ECG and <90 min door-to-PCI [76].

These delays in diagnosis and PCI increase the risk of major adverse cardiac events and may lead to long-term reductions in quality of life [77,78,79].

The mortality rate of in-hospital young patients suffering from AMI is between 0.7% and 7%, typically lower than of older people; however, in the short-term (30 days), young patients without standard risk factors have a 30-times higher risk mortality. A cohort study found that the mortality rate is 120% higher in patients with STEMI compared with NSTEMI, where the risk is 12-times higher [5,80].

In terms of one-year mortality, patients under 40 have similar all-cause mortality rates compared to those aged 41–50 [80]. The mortality rates of STEMI and NSTEMI differ from those of short-term risks: STEMI patients have a two-times higher risk of mortality compared to the general population, and NSTEMI patients have a 2.5–2.8-times higher risk of mortality [5,75,76,77,78,79,81,82,83].

## 7. Atherothrombotic Pathways

**Figure 4:**
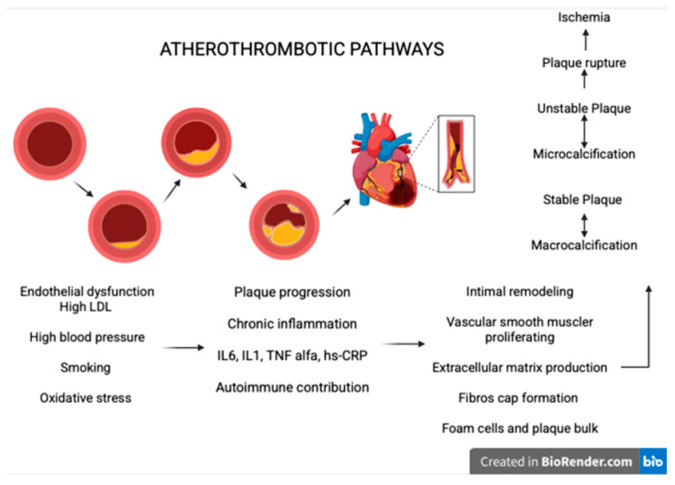
Mechanism of atherosclerosis in AMI; created with BioRender.com. version 4.0.

The classic mechanism of atherosclerosis in AMI involves the following (Figure 4):

1. Endothelial dysfunctions and the response to the injury [84]: The main factors that lead to this process include high LDL cholesterol, elevated systolic blood pressure, smoking, and oxidative stress. These factors increase endothelial permeability, allowing LDL particles to penetrate the intima, where they undergo oxidation and trigger inflammation. At this stage, macrophage apoptosis contributes to further inflammation and endothelial dysfunction [85].
2. Plaque progression: Chronic inflammation sustained by cytokines, represented by IL 6, IL 1, TNF alpha, and hs-CRP, promotes the growth and destabilization of plaque. Autoimmune diseases may contribute to this inflammatory process and accelerate plaque development in young individuals [86,87].
3. Intimal remodulating: The vascular smooth muscle cells respond to inflammation by proliferating and producing extracellular matrix leading to fibrous cap formation and by transforming into foam cells contributing to plaque bulk and potentially necrotic formation [88].
4. Calcification and plaque stability: Unstable plaques are typically characterized by microcalcifications, whereas stable plaques tend to show macrocalcifications. This distinction explains why unstable plaques rupture more frequently, promoting ischemic events [89].

## 8. Non-Atherosclerotic Mechanism

MINOCA is defined as AMI without >50% obstruction of the epicardial coronary artery and represents a common cause of ischemic events, particularly in young people, especially women. It is considered a syndrome rather than a single event, as it encompasses coronary plaque disruption and erosion, epicardial spasm, spontaneous coronary artery dissection (SCAD), and microvascular dysfunction [90]. Long-term studies in young patients reveal a 12% mortality rate over a seven-year follow-up, along with a <30% reduction in the ventricular ejection fraction among young people who suffer from MINOCA [91].

1. SCAD primarily affects young women, often without traditional risk factors (23–36% in women suffering from AMI). In SCAD, the most common mechanism involves an intimal tear that allows blood to enter the media, or rupture of the vasa vasorum leading to intramural hematoma. These two events compress the true lumen of the vessel, producing luminal obstruction. SCAD is associated with fibromuscular dysplasia (pregnancy-related vascular changes, connective tissue disorders, and emotional or physical stressors). The main treatment strategy is conservative management (aspirin + beta-blockers), and the PCI strategy is reserved only for high-risk patients [92,93,94].
2. Vasospasm is a very frequent cause of MINOCA in young people, affecting 20% of them. The main mechanism involves automatic overstimulation, endothelial dysfunction, oxidative stress, and hyperreactivity of smooth muscle contractility. The main treatment strategy is represented by calcium channel blockers and nitroglycerin, avoid beta-blockers, and lifestyle intervention [95].
3. Microvascular dysfunction can result from distal thrombus embolization during PCI, ischemia–reperfusion injury (leading to endothelial swelling, pericyte contraction, glycocalyx shedding, and capillary obstruction), or from microvascular inflammation and oxidative stress, which promote chronic dysfunction and remodeling. Diagnostic approaches include invasive measurements such as the index of microvascular resistance, hyperemic microvascular resistance, and resistance reserve ratio, as well as non-invasive tools like PET imaging to detect obstruction and quantify flow reserve. Management strategies include beta-blockers, calcium channel blockers, ACE inhibitors/ARBs, statins, SGLT-2 inhibitors, and colchicine [4,95,96].
4. Myocardial Infarction in Pregnancy and Postpartum.

Pregnancy-associated myocardial infarction (PAMI) is rare, with an estimated incidence of 3–10 per 100,000 deliveries, but it represents a clinically important cause of maternal morbidity and mortality. The majority of cases occur in women younger than 55 years, making this entity highly relevant in the context of premature MI.

Unlike the atherosclerotic mechanisms that dominate in older adults, the leading causes of PAMI are spontaneous coronary artery dissection (SCAD) and Takotsubo syndrome.

- SCAD accounts for up to 40% of pregnancy-related acute coronary syndromes, particularly in the late third trimester and early postpartum period. Hormonal changes, increased hemodynamic stress, and peripartum vascular remodeling are believed to weaken the arterial wall and predispose to dissection. Management is typically conservative, as most cases heal spontaneously; percutaneous or surgical revascularization is reserved for ongoing ischemia, hemodynamic instability, or left main involvement.
- Takotsubo syndrome is less frequent but has been reported in pregnancy and postpartum, often triggered by emotional or physical stress. It mimics acute coronary syndrome but is characterized by transient left ventricular dysfunction, usually with a favorable recovery. However, its occurrence during pregnancy can complicate maternal and fetal outcomes.

Because of the unique mechanisms and therapeutic considerations, PAMI requires an individualized, multidisciplinary approach involving cardiology, obstetrics, anesthesiology, and neonatology. Long-term follow-up is essential due to the risk of recurrence, particularly in subsequent pregnancies [97].

## 9. Actionable Clinical Recommendations for Preventing Myocardial Infarction in Young Adults (<55 Years)

Although international guidelines do not specify distinct prevention thresholds solely for individuals under 55, several risk factor-specific strategies are especially relevant for this age group due to their extended lifetime risk of cardiovascular disease.

### 9.1. Dyslipidaemia and Lipoprotein(a)

Initiate high-intensity statin therapy as early as possible in all young adults presenting with premature MI or high ASCVD risk. If LDL-C targets remain unmet, add ezetimibe, followed by PCSK9 inhibitors, inclisiran, or bempedoic acid as needed. ESC guidelines now recommend an LDL-C target of <55 mg/dL (1.4 mmol/L), along with at least a 50% reduction from baseline in very-high-risk patients and an even lower target of <40 mg/dL for recurrent ASCVD events [98].

Measure lipoprotein(a) at least once in a lifetime. Elevated Lp(a) levels should prompt more aggressive LDL-C lowering and consideration of cascade screening [99].

Evaluate young adults with premature MI for familial hypercholesterolemia (FH) and offer family screening when indicated [100].

### 9.2. Smoking and Substance Use

Strongly recommend cessation of smoking and vaping, as these remain major preventable risk factors. Advise against the use of cocaine, amphetamines, and cannabis, which are significantly associated with early-onset MI and should be addressed in preventive advice [101].

### 9.3. Hypertension and Metabolic Risk

Target blood pressure levels of <130/80 mmHg (AHA/ACC) or 120–129 mmHg systolic if tolerated (ESC) [102]. In younger individuals with obesity or diabetes, intensify lifestyle interventions and, where appropriate, utilize GLP-1 receptor agonists and SGLT2 inhibitors, which have demonstrated cardiovascular benefits in recent trials including younger subgroups [103].

### 9.4. Obesity and Lifestyle Interventions

Encourage structured lifestyle modifications, including healthy diet, regular physical activity, and weight management. In individuals with obesity and established cardiovascular disease, add-on therapy with semaglutide (GLP-1 RA, 2.4 mg weekly) significantly reduced MACE by approximately 20%, as shown in the SELECT trial [104].

### 9.5. Psychosocial Factors

Screen young adults for depression, anxiety, and psychosocial stressors, as these are recognized contributors to early cardiovascular risk; referral for appropriate care is warranted [105].

## 10. Conclusions and Future Directions

Young adults experiencing AMI represent a distinct clinical subgroup in whom classical cardiovascular risk factors often intersect with unconventional and age-specific contributors such as genetic predispositions, psychosocial stress, systemic inflammation, autoimmune disease, and recreational drug use. Unlike older populations, these patients frequently present with non-obstructive mechanisms—including vasospasm, SCAD, microvascular dysfunction, and MINOCA—leading to greater clinical heterogeneity, atypical presentations, and delays in diagnosis.

Recognizing these distinct mechanisms is not only of academic interest but has direct clinical implications. It underscores the need for tailored prevention strategies and personalized therapeutic approaches that go beyond the traditional paradigm of coronary artery disease in older adults. Early lifestyle interventions, systematic screening for metabolic and psychosocial risk, and the integration of genetic and biomarker data into age-adapted risk models could significantly improve early detection and long-term outcomes.

Ultimately, MI in young adults should not be regarded as a premature form of an “old disease,” but rather as a multi-dimensional syndrome rooted in biology, behavior, and social context. By explicitly acknowledging this complexity, clinicians and researchers can move toward more mechanism-oriented, patient-centered strategies—from calcium channel blockers for vasospasm, to immunomodulation in autoimmune-mediated cases, to psychological support in stress-driven presentations. Such a personalized framework offers the best path to reducing the growing cardiovascular burden in young adults and directly answers the critical question of “so what?”. These insights can and should reshape how we predict, prevent, and manage AMI in younger populations.

## Figures and Tables

**Table 1:** Traditional vs Emerging Risk Factors.

Traditional Risk Factors	Emerging Risk Factors
Dyslipidemia	Recreational drug use (cocaine, cannabis)
Hypertension	Systemic inflammation and autoimmune disease (SLE, RA, psoriasis, vasculitis)
Smoking	Hereditary conditions (hypercolesterolemia and LpA)
Obesity	Psyschosocial factors (stress, depression, burnout)
Diabetes Mellitus	Endothelial dysfunctions and microbiome

SLE—Systemic lupus eerythematosus; RA—Rheumatoid arthritis; LpA—Lipoprotein A.

## Data Availability

All data is available upon request.

## Abbreviations

LDL-C	Low density lipoprotein cholesterol
HDL-C	High density lipoprotein cholesterol
HR	Hazard Ration
CI	Confidence Interval
NSAID	Non-steroidal anti-inflammatory drug
ASCVD	Atherosclerotic cardiovascular disease
ACS	Acute coronary syndrome
TIMI	Thrombolysis in myocardical infarction flow
PCI	Percutaneous coronary intervention
ECG	Electrocardiogram
STEMI	ST-Elevation myocardical infarction
NSTEMI	Non-ST- Elevation myocardical infarction
ACE	Angiotensin-Converting Enzyme
ARBS	Angiotensin II receptor blockers
AHA	American heart association
ACC	American college of cardiology
GLP-1	Gluconat-Like peptide-1
MACE	Major adverse cardiovascular events
MI	Myocardical infarction
CAD	Coronary artery disease
MINOCA	Myocardical infarction with non-obtrustive coronary arteries
CHD	Coronary heart disease
AMI	Acut myocardical infarction
BMI	Body mass index
SID	Systemic inflammation
RA	Rheumatoid arthritis
CV	Cardiovascular
SLE	Systemic lupus eerythematosus
AS	Ankylosing spondylitis
hsCRP	High-sensitivity C-reactive protein
MPO	Myeloperoxidase
Lp-PLA2	Lipoprotein-associated phospholipase A2
TMAO	Trimethylamine-N-oxide
LpA	Lipoprotein A
SCAD	Spontaneous coronary artery dissection
